# Supplementary figures and images for: Microglial FABP4-UCP2 Axis Modulates Neuroinflammation and Cognitive Decline in Obese Mice
Source: Int J Mol Sci. 2022 Apr 14;23(8):4354. doi: 10.3390/ijms23084354 (PMC9032181; doi:10.3390/ijms23084354)

## Total Distance

**A.**

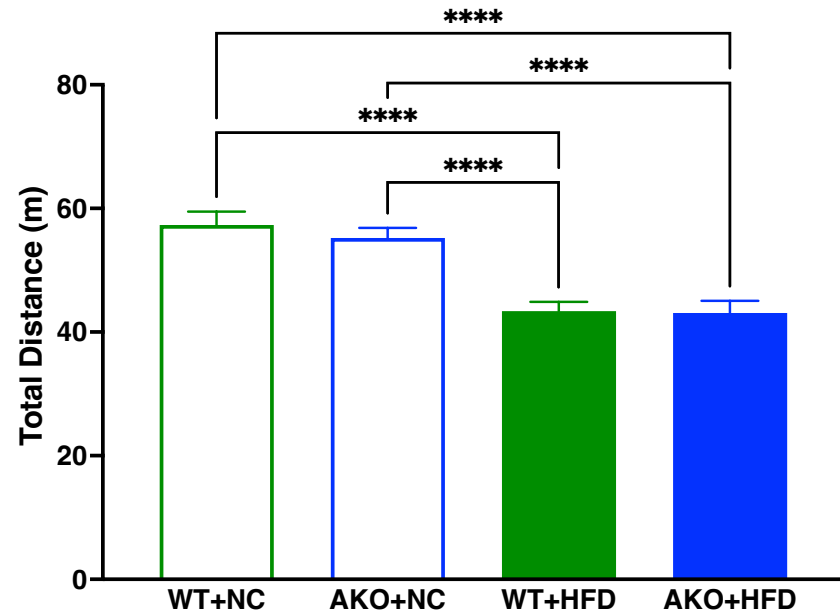

## Average Velocity

**B.**

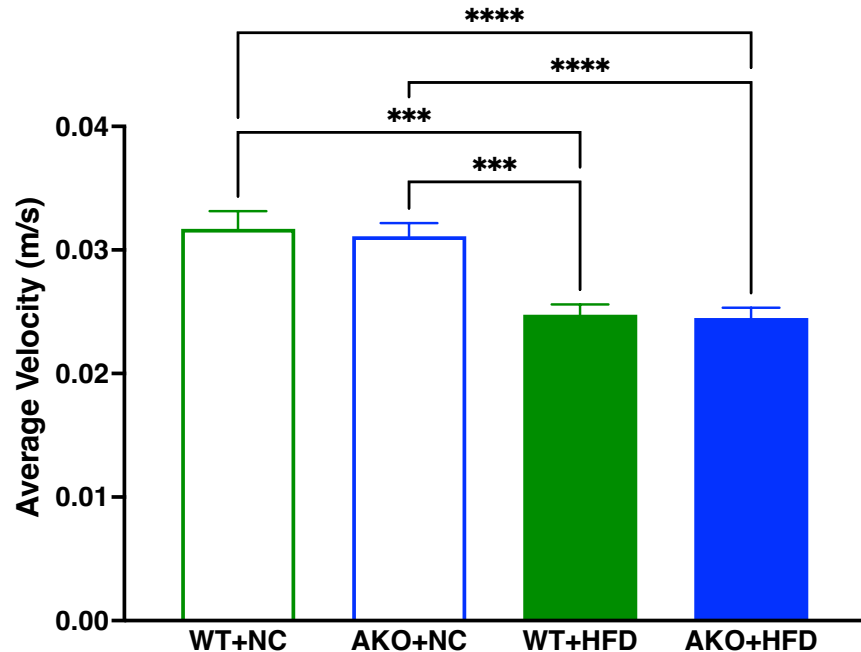

Supplement: Supplementary file 1 [file ijms-23-04354-s001.zip › So et al., 2022 Figure S1.pdf]

**A.****Inner Time**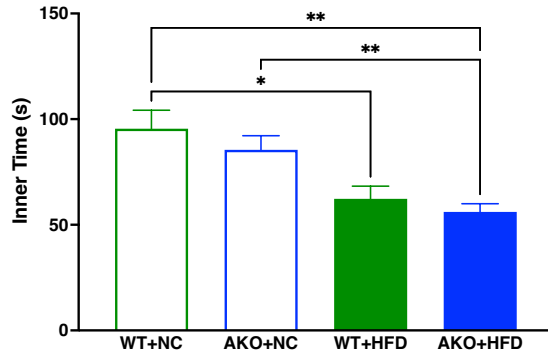**B.****Outer Time**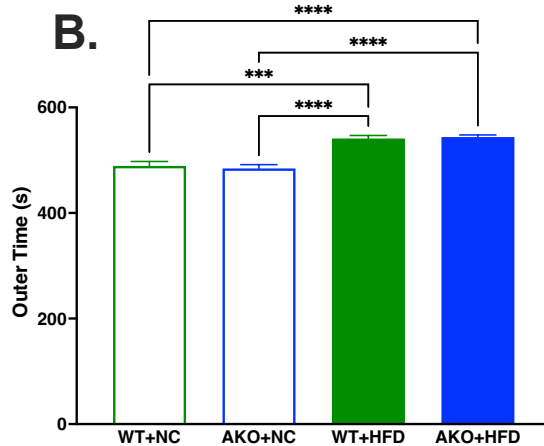**C.****Inner/Outer Ratio**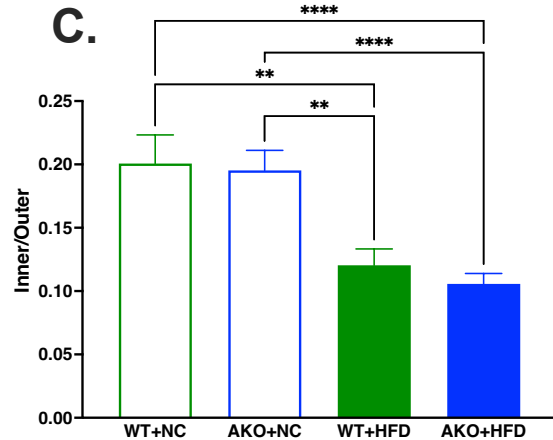

Supplement: Supplementary file 1 [file ijms-23-04354-s001.zip › So et al., 2022 Figure S2.pdf]
